# Liprin-α1 and ERC1 control cell edge dynamics by promoting focal adhesion turnover

**DOI:** 10.1038/srep33653

**Published:** 2016-09-23

**Authors:** Veronica Astro, Diletta Tonoli, Sara Chiaretti, Sabrina Badanai, Kristyna Sala, Marino Zerial, Ivan de Curtis

**Affiliations:** 1 Cell Adhesion Unit, Division of Neuroscience, IRCSS San Raffaele Scientific Institute and San Raffaele University, via Olgettina 58, 20132 Milano, Italy; 2 Max-Planck-Institute of Molecular Cell Biology and Genetics, Pfotenhauerstr. 108, 01307 Dresden, Germany

## Abstract

Liprin-α1 and ERC1 are interacting scaffold proteins regulating the motility of normal and tumor cells. They act as part of plasma membrane-associated platforms at the edge of motile cells to promote protrusion by largely unknown mechanisms. Here we identify an amino-terminal region of the liprin-α1 protein (liprin-N) that is sufficient and necessary for the interaction with other liprin-α1 molecules. Similar to liprin-α1 or ERC1 silencing, expression of the liprin-N negatively affects tumor cell motility and extracellular matrix invasion, acting as a dominant negative by interacting with endogenous liprin-α1 and causing the displacement of the endogenous ERC1 protein from the cell edge. Interfering with the localization of ERC1 at the cell edge inhibits the disassembly of focal adhesions, impairing protrusion. Liprin-α1 and ERC1 proteins colocalize with active integrin β1 clusters distinct from those colocalizing with cytoplasmic focal adhesion proteins, and influence the localization of peripheral Rab7-positive endosomes. We propose that liprin-α1 and ERC1 promote protrusion by displacing cytoplasmic adhesion components to favour active integrin internalization into Rab7-positive endosomes.

Cell migration and invasion require the coordination of adhesion, cytoskeletal reorganization and membrane traffic to promote the protrusive activity at the front of the migrating cells^1^. An important question is how these processes are coordinated. Complex molecular networks are expected to be involved, and may become specific targets to interfere with the metastatic potential of invasive tumor cells. Others and we have shown that the scaffold protein liprin-α1 is required for efficient migration and tumor cell invasion *in vitro* and *in vivo*^2^,^3^. Liprin-α1 can dimerize and bind to different protein partners, and may thus contribute to the formation of large supramolecular assemblies linking together structural and signaling proteins^4^,^5^. The adaptors ERC1 (also known as ELKS, Rab6 Interacting Protein, CAST)^6^ and LL5^7^ act as coplayers of liprin-α1 in the regulation of cortical platforms at the cell periphery, where the 3 proteins collaborate to promote migration and invasion^8^,^9^,^10^. During migration the 3 proteins colocalize near the protruding front of migrating cells, where they identify novel dynamic and polarized cytoplasmic structures that disappear when protrusion halts^10^. The data so far indicate that the 3 proteins stabilize the edge of migrating tumor cells, and regulate the internalization of active integrin receptors. We have recently proposed a model in which liprin-α1 and ERC1 are part of dynamic plasma membrane–associated platforms (PMAPs) important for the polarized migration of tumor cells during invasion^11^.

It is known that the amino-terminal coiled-coil region of liprins is needed for homo-dimerization: the fragment including amino acid residues 1 to 670 is sufficient to form homodimers, while deletion of the first 793 amino-terminal residues prevents homodimerization^12^. Here, we have identified a shorter amino-terminal region of liprin-α1 (residues 1–344 = liprin-N mutant) that is necessary and sufficient for homodimerization, and we have analyzed the effects of liprin-N and liprin-ΔN (missing residues 1–334) mutants and of protein silencing on cell motility and focal adhesion dynamics at the front of cells migrating on fibronectin. We found that liprin-N interferes with tumor cell migration and invasion *in vitro*. These effects are due to interference with the localization of ERC1 at the cell edge, where ERC1 is required for focal adhesion turnover. Distinct clusters of active β1 integrins colocalized either with ERC1 or with focal adhesions at the cell edge, where ERC1 influenced the localization of Rab7-positive endosomes. Based on these findings, we propose a model for the liprin-α1–, ERC1–mediated disassembly of focal adhesions during cell migration.

## Results

### Identification of an amino-terminal region required for the homodimerization of liprin-α1

**Figure 1:**
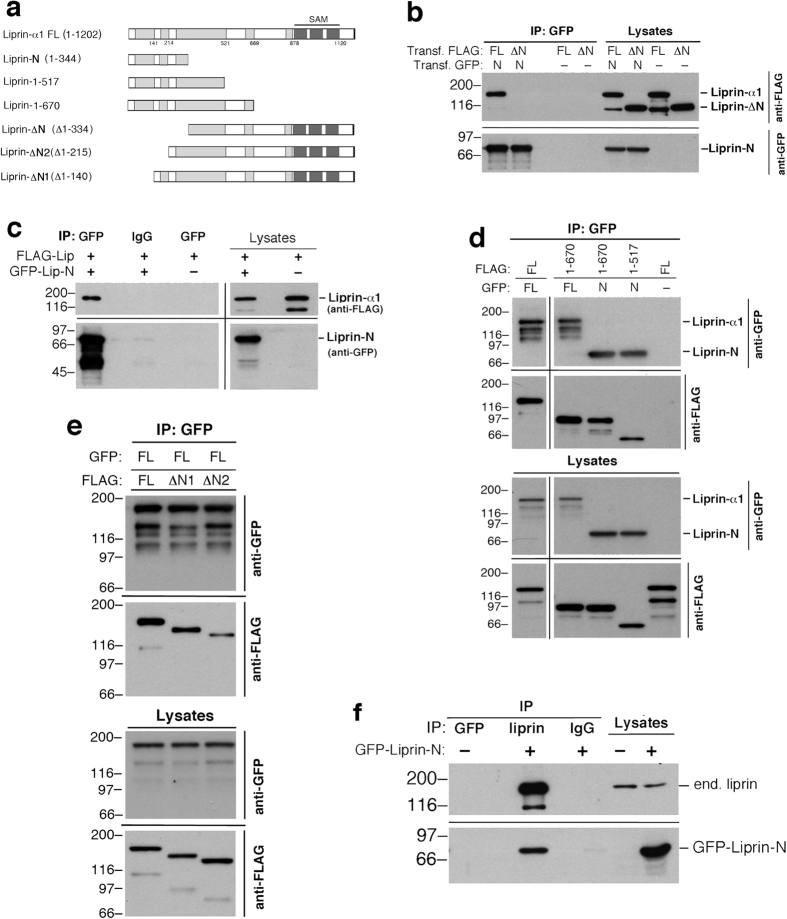
The amino-terminal region of liprin-α1 is necessary and sufficient for the formation of homo-complexes. (**a**) Scheme of the constructs used in this study. In (**b**–**f**) COS7 cells cotransfected with the indicated GFP-tagged and/or FLAG-tagged constructs were lysed for immunoprecipitation with the indicated antibodies; 300 μg of protein lysate were used for each immunoprecipitation; lanes with lysates were loaded with 30 μg of protein. (**b**) Filters from immunoprecipitations with anti-GFP antibody (IP, left) and from lysates (right). GFP-Liprin-N (N) interacts with full length FLAG-Liprin-α1 (FL), but not with FLAG-Liprin-ΔN (ΔN). Control lanes were loaded with immunoprecipitates from lysates of cells transfected only with FLAG-Liprin constructs. (**c**) Left: immunoprecipitations (IP) with anti-GFP (GFP) or control IgG (IgG) from lysates of cells cotransfected with FLAG-Liprin-α1 (FLAG-Lip) and GFP-Liprin-N (GFP-Lip-N), or with FLAG-Liprin-α1 only. Right: blots from gels loaded with aliquots of lysates. (**d**,**e**) Filters with immunoprecipitations (IP, top) and aliquots of lysates (bottom) from cells cotransfected/transfected with the indicated GFP- and/or FLAG-tagged constructs were incubated with anti-FLAG antibodies, then stripped and re-incubated with anti-GFP antibodies. (**f**) Lysates from cells transfected with GFP-Liprin-N or from non-transfected cells were immunoprecipitated (IP) with anti-GFP, anti-liprin-α1, or control IgG; aliquots of the two lysates used for IP are shown on the right. FL, full length liprin-α1; N, liprin-N; ΔN, liprin-ΔN; ΔN1, liprin-ΔN1; ΔN2, liprin-ΔN2; 1–670, Liprin-1-670; 1–517, Liprin-1-517. Blots in panels (c,d) have been cropped, and full blots are presented in Supplementary Fig. S8a.

To address the role of liprin-α1 dimerization in cell migration we have first defined a region required for the formation of liprin-α1 homo-complexes. We have prepared the liprin-α1 mutant GFP-Liprin-N including the first half (amino acids 1–344) of the amino-terminal region of liprin-α1 (amino acids 1–670) previously identified as needed for homodimerization^12^ (Fig. 1a). Co-immunoprecipitation experiments showed that liprin-N interacts specifically with the full length FLAG-liprin-α1 protein (Fig. 1b). This result indicates that liprin-N is sufficient to form homo-complexes with other liprin-α1 molecules. In parallel we tested the coimmunoprecipitation of GFP-Liprin-N with the FLAG-liprin-ΔN mutant lacking the amino-terminal fragment involved in homodimerization. Deletion of the amino-terminal fragment completely abolished the interaction with GFP-Liprin-N, indicating that this fragment is necessary for homodimerization (Fig. 1b,c). The liprin-N fragment can specifically oligomerize also with fragments of liprin-α1 including the liprin-N region (Fig. 1d). Mutants liprin-ΔN1 and liprin-ΔN2 that include deletions of the amino-terminal region shorter than in liprin-ΔN could still homodimerize to some extent with the full length protein (Fig. 1e). These results suggest that an extended region within liprin-N is required for homo-dimerization. Moreover, liprin-N interacted specifically with endogenous liprin-α1 protein, suggesting that it may interfere with the function of the endogenous liprin complex (Fig. 1f).

### Liprin-N is required for efficient cell motility and invasion

**Figure 2:**
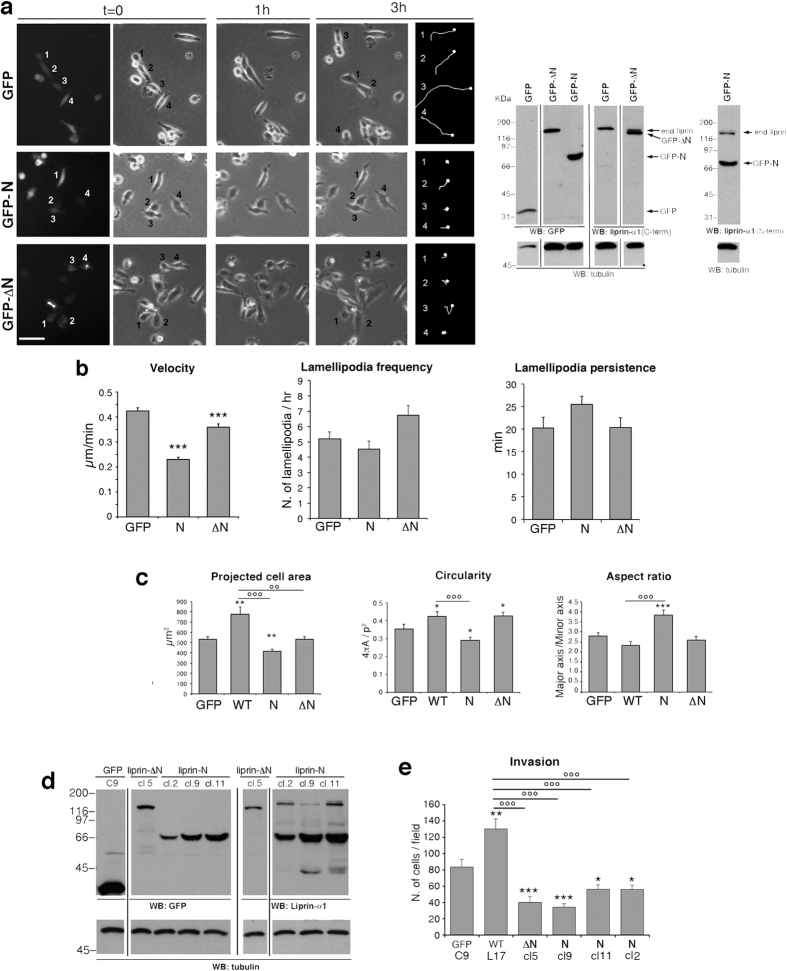
Liprin-N interferes with tumor cell motility and invasiveness. (**a**) Frames from time-lapse of MDA-231 cells transfected with GFP-tagged constructs. Cells were visualized after transfection on fibronectin-coated substrates. Numbers indicate transfected GFP-positive cells (left panel), at the beginning and end of 3 h monitoring. The last column on the right shows the tracks (3 h) of the cells indicated by respective numbers on the left (NB: tracks are oriented differently from the cells shown in the time frames). Scale bar, 50 μm. Right: blots for relative levels of transfected constructs with respect to endogenous liprin-α1: intensity of GFP-Liprin-N and GFP-Liprin-ΔN were respectively about 4-fold and 6-fold stronger than endogenous liprin-α1. (**b**) MDA-231 cells transfected with the indicated constructs were quantified for speed of migration (left; n of cells is 469 for GFP; 398 for liprin-N; 434 for liprin-ΔN); for frequency (centre) and duration (right) of lamellipodia (n = 15 cells per condition). (**c**) Influence of liprin constructs on the morphology of transfected cells freely migrating on fibronectin. Projected cell area (left graph), circularity (centre) and aspect ratio (right) were measured as described in the Methods (n = 60–66 cells per experimental condition). In the right graph, **A** is the projected cell area, **p** is the cell perimeter. (**d**) Immunoblotting with anti-GFP (left), anti-liprin-α1 (right), and anti-tubulin (bottom) antibodies on lysates from the indicated cell clones obtained by transfection and selection of MDA-231 cells with the indicated constructs. Right blot: two different anti-liprin-α1 antibodies were used: the antibody used for the left filter (from Santa Cruz) was less efficient, and used to identify liprin-ΔN that lacks the epitope recognized by the antibody from Proteintech, used on left filter. (**e**) Matrigel invasion assays performed with cell lines stably expressing the indicated constructs, as detailed in the Methods. Bars in (**b**,**c**,**e**) are means ± s.e.m.; significant differences detected by the Student t-test: * and °*p* < 0.05; ** and °°*p* < 0.005; *** and °°°*p* < 0.0005; asterisks (*) refer to differences with respect to GFP-transfected cells; circles (°) refer to differences with respect to GFP-liprin-α1 (WT). Blots in (**a**,**d**) are cropped; see full blots in Supplementary Fig. S8b.

MDA-231 breast cancer cells plated on fibronectin were transfected with the indicated GFP-tagged constructs, and monitored during free migration (Fig. 2a). The speed of migration was significantly reduced in cells expressing liprin-N, and more weakly affected by the expression of the deletion mutant liprin-ΔN (Fig. 2b). Migration is driven by the formation of lamellipodia at the cell front. We evaluated the protrusive activity of the cells expressing the different GFP-tagged constructs. The frequency of lamellipodia formation was not affected by the expression of liprin-N and was mildly increased by the expression of liprin-ΔN. Moreover, neither mutant influenced the persistence of the lamellipodia. Therefore although the migration is impaired, the formation of stable lamellipodia *per se* was not affected in cells transfected with mutants interfering with the formation of endogenous liprin-α1 dimers. We tested whether the milder effects observed after expression of liprin-ΔN compared to liprin-N was due to the presence of the endogenous liprin-α1 protein by transfecting the plasmids for the GFP-Liprin-ΔN mutant together with the siRNA for liprin-α1. The results show that even after silencing the endogenous protein, GFP-Liprin-ΔN had only minor effects on migration on fibronectin (Supplementary Figs 1 and 2).

By looking at the shape of the cells, while GFP-liprin-α1 overexpressing cells had an increased projected cell area compared to GFP expressing cells, as previously reported^2^, expression of GFP-Liprin-N significantly reduced the projected cell area and induced an elongated cell shape, confirmed by measuring the circularity and the aspect ratio of the cells (Fig. 2c). GFP-Liprin-ΔN did not alter the projected cell area compared to control GFP-transfected cells, but prevented the increase in spreading observed after overexpression of full length GFP-Liprin-α1.

We next measured the ability of the liprin-α1 mutants to interfere with the invasive potential of MDA-231 tumor cells *in vitro*. The functional complex including liprin-α1 and ERC1 is needed for efficient invasion *in vitro*. Liprin-α1 overexpression increases the invasive potential measured by Matrigel invasion assays, while liprin-α1 depletion strongly impairs invasion *in vitro* and *in vivo*^2^,^3^,^10^. To measure invasion, we produced MDA-231 cell lines stably transfected with GFP-Liprin-N or GFP-Liprin-ΔN, to be compared with control GFP cell lines (Fig. 2d). Analysis of cell proliferation by MTT assays revealed no significant differences among the growth rates of GFP-Liprin-N and GFP-Liprin-ΔN cell lines compared to GFP-expressing or wild type MDA-231 cells (Supplementary Fig. S3). Matrigel transwell assays demonstrated that while full length GFP-Liprin-α1 potentiated invasion, both GFP-Liprin-N (3 independent clones) and GFP-Liprin-ΔN strongly inhibited invasion (Fig. 2e). Given its ability to mediate the formation of homo-complexes (Fig. 1), liprin-N may inhibit cell motility and invasion by acting as a dominant negative that interacts with endogenous liprin-α1. Liprin-N may interfere with the function of endogenous liprin-α1 by forming mixed liprin-N/endogenous liprin-α1 dimers (Fig. 1), thus possibly interfering with the normal function of the endogenous complexes including liprin-α1 and its interacting partners. How liprin-ΔN inhibits invasion (and only poorly migration on fibronectin) is less obvious. One possibility is that liprin-ΔN plays a negative effect on motility by binding endogenous liprin-interacting partners, thus preventing them from binding to endogenous liprin-α1.

### Liprin-N interferes with the localization of ERC1 at the protruding edge of migrating cells

**Figure 3:**
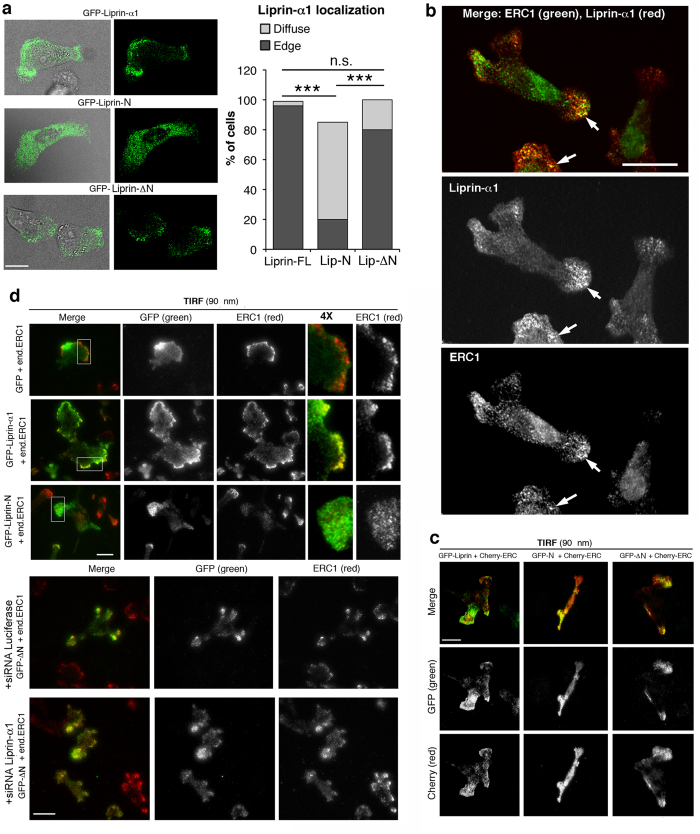
Effects of different liprin-α1 constructs on the subcellular localization of ERC1. (**a**) Laser scanning confocal microscope images of MDA-231 cells plated on fibronectin. Left is a merge of the confocal image (green) and phase contrast to show the shape of the cells transfected with the indicated GFP-tagged liprin-α1 construct. Graph on the right: quantification of transfected cells with diffuse (GFP-positive) signal or with signal concentrated at the cell edge (n = 31–49 cells from 3 independent experiments). ***P < 0.0001 by the χ^2^ test. (**b**) Cells were seeded on fibronectin-coated coverslips, fixed and processed for immunofluorescence to reveal the indicated endogenous proteins. Images were acquired at UltraViewer spinning disk confocal microscope. Scale bar 20 μm. (**c**) TIRF microscopy of cells cotransfected with the indicated GFP-tagged liprin-α1 constructs (green) and mCherry-ERC1 (red). The upper row shows the merge of green (second row) and red (bottom row) channels. Scale bar, 20 μm. (**d**) TIRF microscopy of cells transfected with the indicated GFP-tagged liprin-α1 constructs (green) and siRNAs, immunostained to detect endogenous ERC1 (red). From left: the first column shows the merged TIRF images at the bottom of the cells (90 nm); the second and third rows show the distributions of the transfected liprin-α1 constructs and of endogenous ERC1, respectively; the last two columns to the right show 4-fold enlargements of the protrusions highlighted in the first column. Scale bars, 20 μm.

We have characterized the subcellular localization of the full length and truncated mutants of liprin-α1 by confocal and total internal reflection fluorescence (TIRF) microscopy. Confocal imaging on migrating MDA-231 cells transfected with GFP-tagged liprin constructs showed that while full length liprin-α1 specifically concentrated near the protruding cell edge, liprin-N had a diffuse cytoplasmic distribution (Fig. 3a). On the other hand the homodimerization-defective liprin-ΔN mutant could still partially localize to the cell edge. We have previously shown that endogenous liprin-α1 was required for the localization of ERC1 at the cell edge^10^, where the two endogenous proteins partially colocalize (Fig. 3b). We therefore tested the effects of the expression of liprin-N on the localization of ERC1. As expected, the cotransfection of mCherry-ERC1 with GFP-Liprin-α1 resulted in the colocalization of the 2 tagged proteins at the edge of MDA-231 cells migrating on fibronectin (Fig. 3c). Strikingly, coexpression of mCherry-ERC1 with GFP-Liprin-N resulted in a diffuse localization of both proteins, indicative of the disrupting effect of liprin-N on ERC1 localization, even in the presence of the endogenous liprin-α1. Liprin-N had similar effects on the localization of the endogenous ERC1 protein; in non-transfected cells (not shown) as well as in GFP-transfected cells endogenous ERC1 localized specifically at cell protrusions (Fig. 3d). GFP-Liprin-α1 was concentrated with endogenous ERC1 at the cell periphery, while expression of GFP-Liprin-N strongly affected the distribution of endogenous ERC1 that became more diffuse as GFP-Liprin-N in most cells. This effect is suggestive of the interference of the liprin-N fragment with endogenous liprin-α1 function and with the assembly of a functional complex including endogenous ERC1, supporting the dominant negative effect of liprin-N. The GFP-Liprin-ΔN mutant unable to bind other liprin-α1 molecules could partially localize at the cell edge, and failed to prevent the localization of mCherry-ERC1 at these sites (Fig. 3c), possibly because endogenous liprin-α1 and ERC1a may still interact at the cell periphery^10^. Accordingly, cotransfection of GFP-Liprin-ΔN with a siRNA to silence endogenous liprin-α1 resulted in more evident displacement of endogenous ERC1 from the cell periphery (Fig. 3d). Together, these results show that the amino-terminal liprin-N fragment may have dominant negative effects on the organization of the protruding edge of mobile cells.

### Liprin-N regulates the disassembly of focal adhesions in migrating cells

**Figure 4:**
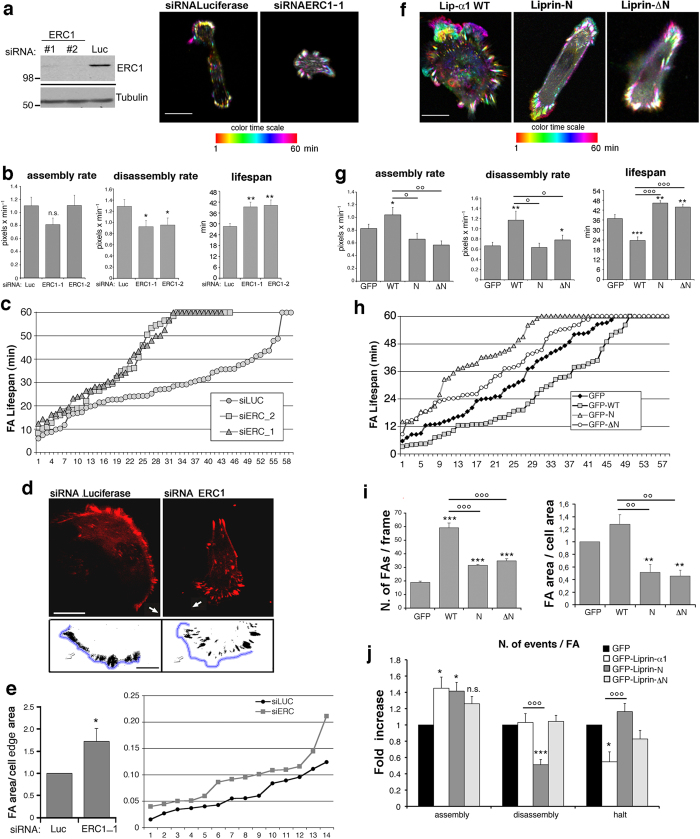
ERC1 and liprin dimerization regulate the morphology and dynamics of peripheral focal adhesions in migrating cells. (**a**) Left: depletion of ERC1 by two siRNAs in lysates (50 μg). Right: Colour-coded maximal-intensity projection from 1 h time-lapse series of mCherry-Zyxin. White indicates static adhesions. Scale bar, 20 μm. Full blots are presented in Supplementary Fig. S8c. (**b**) Bars are means ± s.e.m. (**c**) Focal adhesions lifespan (n = 43–59) from cells prepared as in (**b**). (**d**) Confocal frames from migrating MDA-231 cells (arrows) cotransfected with mCherry-Zyxin and siRNA. Scale bar, 10 μm. Bottom: cell boundaries outlined in blue. Scale bar, 5 μm. (**e**) Silencing of ERC1 decreased the area occupied by adhesions at the cell periphery. The area of protruding cell edge occupied by adhesions was calculated on thresholded 20 × 10 μm peripheral regions. Bars are normalized means ± s.e.m. (n = 14–18 protrusions). On the right is the distribution of the values (n = 14 protrusions per experimental condition). (**f–j**) Liprin-N and liprin-ΔN mutants perturb focal adhesion dynamics. (**f**) Color-coded maximal-intensity projection of the mCherry-Zyxin signal from 1 h acquisition of cells expressing mCherry-Zyxin and GFP-tagged mutants. Scale bar 10 μm. (**g**) Bars are means ± s.e.m. of rate of assembly (n = 32–87 events), disassembly (n = 39–70 events), and lifespan of focal adhesions (n = 60–120 focal adhesions). (**h**) Adhesion lifespan at cell periphery (n = 50–58 adhesions). (**i**) Left: means of focal adhesions/frame (average from different frames; 60 frames per cells; n = 72–177 adhesions from 3–4 experiments). Right: normalized ratio between the area of peripheral focal adhesions and the total cell area (n = 171–296 focal adhesions from 2–3 independent experiments. (**j**) Quantification of the assembly, disassembly, and halt events per focal adhesion during 60 minutes acquisition of MDA-231 cells transfected with the indicated mutants. Bars show means ± s.e.m. normalized to control (GFP) values (n =  60–118 focal adhesions from 3 independent experiments). In (**g**,**i**,**j**) values significantly different from controls (GFP) are indicated by asterisks, while values significantly different from GFP-Liprin-α1 (WT) are indicated by circles. °, *P < 0.05; °°, **P < 0.005; °°°, ***P < 0.0005; n.s., no significant difference with control. Student’s *t*-test.

The dynamics of integrin-mediated adhesion is essential for the protrusive activity of cells migrating on extracellular matrix^13^. We have tested if interfering with the correct subcellular localization of the liprin-α1/ERC1 functional complex may affect the dynamics of focal adhesion turnover at the front of migrating cells. Analysis of the dynamics of focal adhesions in cells cotransfected with two different siRNAs specific for ERC1 and the focal adhesion marker mCherry-Zyxin showed that ERC1 silencing resulted in more static focal adhesions (Fig. 4a). Quantitative analysis showed that silencing of ERC1 specifically inhibited the rate of disassembly of focal adhesions, while the rate of assembly was not affected (Fig. 4b, left and central graphs). As a consequence, the average lifespan of focal adhesions was increased after ERC1 knockdown (Fig. 4b, right graph; Fig. 4c), indicative of a relationship between focal adhesions turnover and the reduced speed of migration. As a consequence of the specific decrease of focal adhesion disassembly rate, the fraction of the protruding cell periphery occupied by focal adhesions was increased after ERC1 silencing (Fig. 4d,e). These data support an important role of ERC1 in driving the turnover of focal adhesions at the edge of cells migrating on extracellular matrix.

Previous findings had shown that liprin-α1 is also required for focal adhesion turnover^10^. Here we analyzed the effects of targeting liprin-α1 homodimerization on focal adhesion dynamics. For this, we analyzed the effects of the liprin-N and liprin-ΔN mutants on the dynamics and morphology of focal adhesions, by time-lapse imaging on MDA-231 cells cotransfected with the focal adhesion marker mCherry-Zyxin together with either GFP, GFP-Liprin-α1, GFP-Liprin-N, or GFP-Liprin-ΔN (Supplementary movies S1 to S4). As observed after depletion of the endogenous ERC1 protein, the expression of either mutant in MDA-231 cells resulted in more static focal adhesions compared to control cells (GFP), and to cells overexpressing GFP-tagged full length liprin-α1 (Fig. 4f). Quantitative analysis confirmed that overexpression of GFP-liprin-α1 enhanced the dynamics of focal adhesions by increasing both rate of assembly and disassembly, while both processes were largely unaffected in cells expressing either liprin-N or liprin-ΔN (Fig. 4g). The lifespan of focal adhesions was decreased upon GFP-liprin-α1 expression, and increased upon either liprin-N or liprin-ΔN expression, showing that both mutants negatively affected focal adhesion dynamics (Fig. 4g, right graph; Fig. 4h). This may be explained by the interference of the two constructs with the function of the endogenous complexes. By binding to endogenous liprin-α1 (Fig. 1), liprin-N may disrupt the normal assembly of functional complexes by endogenous liprin-α1. On the other hand the negative effects on focal adhesion dynamics by liprin-ΔN are probably due to its inhability to assemble into oligomers. GFP-Liprin-α1 strongly increased the number of focal adhesions present in migrating cells and slightly affected their average size. By comparison, only a weak increase in the number of focal adhesions was observed in cells expressing either the GFP-Liprin-N or the GFP-Liprin-ΔN mutant (Fig. 4i).

Altogether these results show that both the liprin-N N-terminal fragment required for homo-dimerization, and the liprin-ΔN deletion mutant unable to homo-dimerize perturb the morphology and dynamics of focal adhesions, with a stronger increase in the lifespan of focal adhesions induced by liprin-N. Although the expression of liprin-N did not reduce the rate of disassembly, it strongly reduced the number (frequency) of disassembly events (Fig. 4j). The data show that the increase in focal adhesion dynamics observed upon overexpression of the full length liprin-α1 was completely abolished when the homodimerization domain was missing (liprin-ΔN mutant), but also when only the fragment needed for homodimerization (liprin-N mutant) was expressed (Fig. 4g,h). The results suggest that liprin-N interferes with the dynamics of focal adhesions by preventing the correct recruitment of endogenous liprin-associated complexes at the leading edge of the migrating cells, and that ERC1 localization in proximity of focal adhesions is required to mediate its function on focal adhesion disassembly.

### Differential colocalization of active β1 integrins with either vinculin or ERC1/liprin-α1

**Figure 5:**
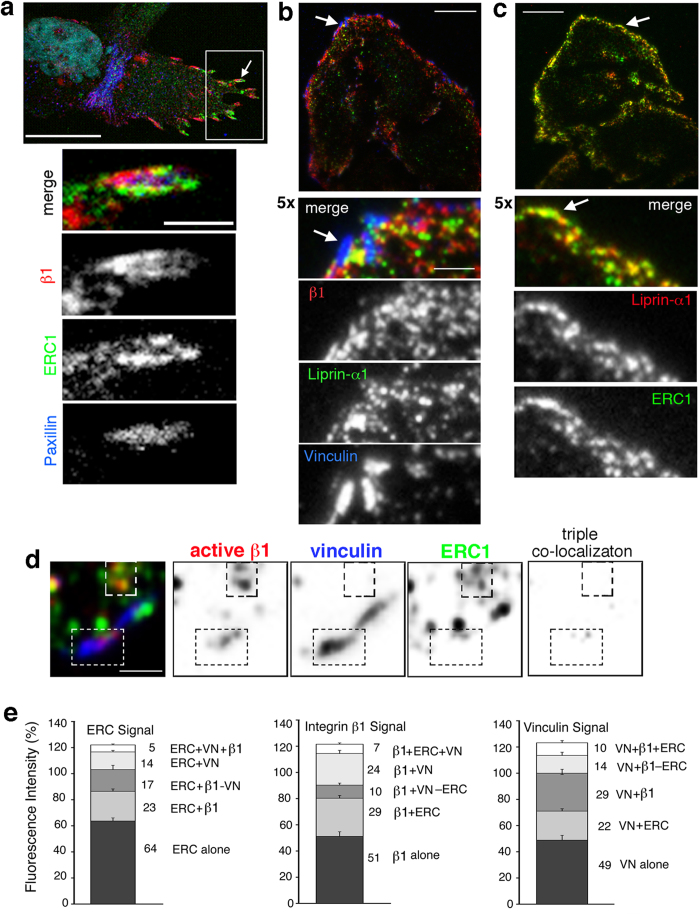
Colocalization of ERC1 with active integrins at sites lacking cytoplasmic focal adhesion proteins. (**a**) Confocal microscopy showing the immunolocalization of endogenous active β1 integrins (by mAb 9EG7), ERC1, and paxillin. Scale bar, 20 μm. The white arrow indicates the focal adhesion area enlarged in the images underneath. Scale bar, 2 μm. (**b**,**c**) TIRF images of HeLa cells immunostained for the indicated endogenous proteins. Scale bar, 20 μm. White arrow indicates the area shown in the 5-fold enlargement underneath. Scale bar, 4 μm. (**d**) Example of a TIRF image from the peripheral area of an MDA-231 cell on fibronectin used for the analysis of the colocalization between endogenous active β1 integrins, focal adhesion marker vinculin, and ERC1. The 3 channels from the merge on the left are shown as reversed images showing the localization of each protein. The last panel on the right shows the distribution of the triple colocalization within the selected area. Dotted rectangles show two examples of areas with little or no triple colocalization. Scale bar 2 μm. **(e**) Quantitative analysis of fluorescence intensity in the proximity of vinculin-positive focal adhesions on TIRF images of MDA-231 cells. Representations of the single, double and triple localization of the 3 markers (vinculin, ERC1, active β1) with respect to total ERC1 (left graphs), total active β1 (central graphs), and total vinculin signal (right graphs); n = 22 focal adhesions from 4 cells.

Our data indicate that liprin-α1 affects the turnover of focal adhesions by recruiting ERC1 at the protruding cell edge (Figs 3 and 4). High resolution confocal and TIRF imaging showed the partial colocalization of active β1 integrins identified by the 9EG7 mAb with focal adhesions detected by anti-vinculin and anti-paxillin antibodies. Active β1 partially colocalized also with endogenous ERC1 at the plasma membrane of different cell types, such as MDA-231 and HeLa cells (Fig. 5a,b). Interestingly, it was evident that the areas of colocalization of active β1 with either focal adhesion markers or ERC1 were distinct in many cases (Fig. 5a,b, 5x enlargements). As previously shown by others and us, the subcellular localization of liprin-α1 and ERC1 largely colocalized near focal adhesions at the cell edge (Fig. 5c), indicating that integrin β1 partially colocalized with the liprin-α1–ERC1 complex at these sites. The quantification of the differential colocalization of active β1 with either ERC1 or focal adhesions was performed as detailed in the Methods, on selected areas around vinculin-positive focal adhesions. Notably, the triple colocalization among the 3 proteins was often very limited or absent (Fig. 5d). Quantification performed at/near focal adhesions confirmed the differential colocalization of active β1 integrins with either endogenous ERC1 or the focal adhesion components vinculin (Fig. 5e) and paxillin (not shown). Therefore a significant fraction of active β1 colocalized with ERC1 at sites where no focal adhesion proteins were present.

### ERC1 silencing specifically affects the localization of Rab7-positive endosomes at the cell periphery

**Figure 6:**
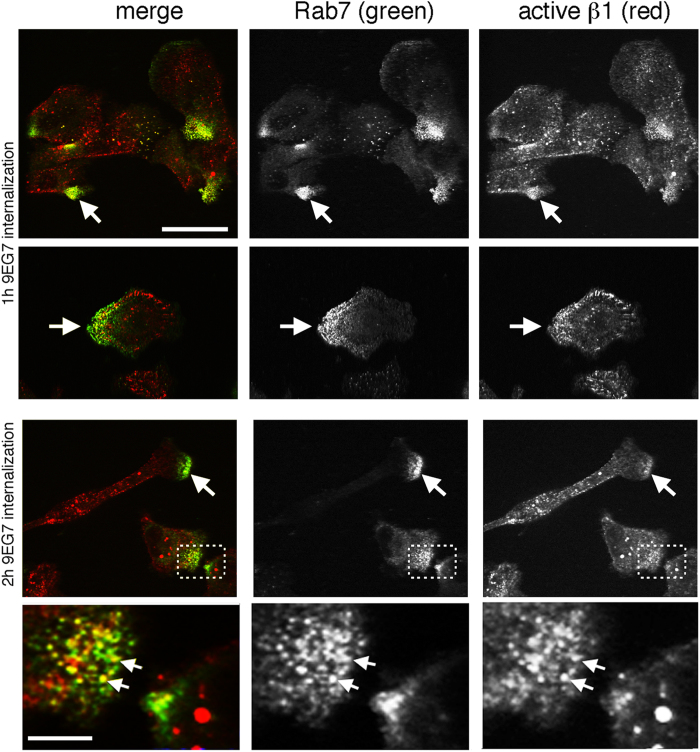
Colocalization of Rab7 with internalized active integrins at protrusions. Confocal microscopy of MDA-231 cells plated on fibronectin and incubated for 1 or 2 h with 1.25 μg/ml of 9EG7 mAb recognizing active β1 integrins. After fixation, cells were immunostained for endogenous Rab7, and then with secondary antibodies to reveal also the internalized 9EG7 mAb. Scale bars, 20 μm. White arrows and the dotted rectangle show examples of protrusions, enlarged in the image underneath, where the overlap between Rab7 and active integrin signals is evident. Scale bar 5 μm.

**Figure 7:**
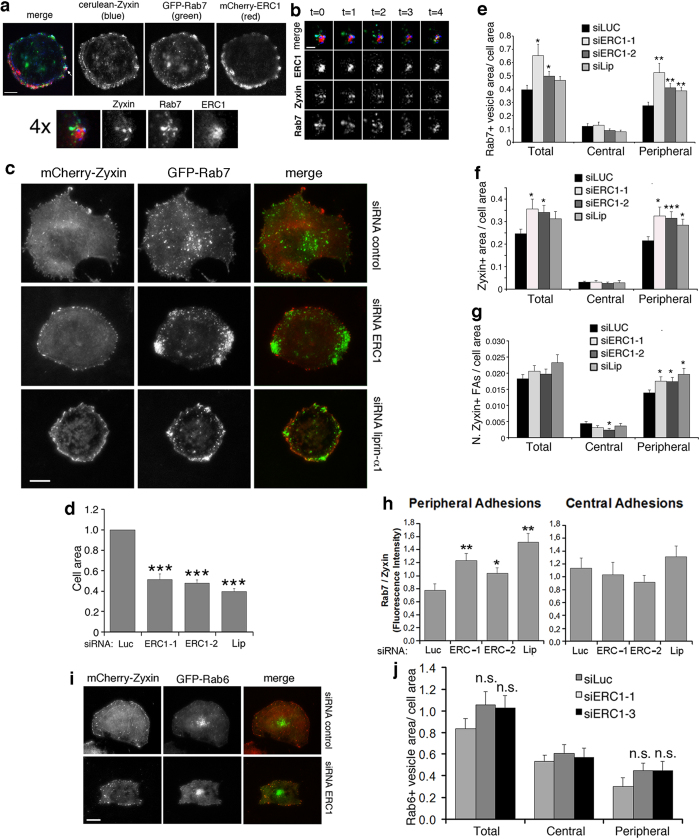
ERC1 and liprin-α1 regulate the distribution of focal adhesions and Rab7-positive endosomes at the cell periphery. (**a**) Frame from a time-lapse (TIRF, see Supplementary movie S5) of COS7 cell cotransfected with GFP-Rab7, mCherryERC1 and cerulean-Zyxin. Below, 4-fold enlargement of the area indicated by the arrow: the composite distribution of the 3 markers around the zyxin-positive focal adhesions is shown. Scale bar, 20 μm. (**b**) Frames from time lapse at the TIRF microscope (see Supplementary movie S6) from the peripheral area of a cell transfected as in (**a**), showing the dynamic behavior of the 3 markers. Scale bar, 4 μm. (**c**) COS7 cells were plated on fibronectin, cotransfected with GFP-Rab7, mCherry-Zyxin and indicated siRNA. TIRF images were used for quantification. Scale bar, 20 μm. (**d**) Projected cell area (n = 35–41 cells from 3 independent experiments) normalized on control cells (siLuc). (**e**) Ratio between Rab7-positive area and projected cell area (pixels/pixels). (**f**) Ratio between zyxin-positive focal adhesion area and projected cell area (pixels/pixels). (**g**) Ratio between number of zyxin-positive focal adhesions and projected cell area (n = 36–43 from 3 experiments). (**h**) Intensity of Rab7-positive signal per focal adhesion at cell periphery (n = 28–41) or in central area of the cell (n = 17–28). (**i**) TIRF images of COS7 cells cotransfected with mCherry-Zyxin, GFP-Rab6, and siRNAs. Scale bar, 20 μm. (**j**) Ratio between Rab6-positive area and projected cell area (pixels/pixels) (n = 34–41 from 3 experiments). Bars in (**d**–**h**,**j**) are normalized means ± s.e.m. *P < 0.05; **P < 0.005; ***P < 0.0005; n.s., no significant difference with control.

Integrin internalization and recycling contributes to the assembly and disassembly of focal adhesions^14^, and we found that ERC1 and liprin-α1 regulate the internalization of active β1 integrin^10^. In MDA-231 cells active β1 integrins are specifically targeted to Rab7-positive endosomes^15^, which are involved in focal adhesion turnover^16^. We confirmed that internalized 9EG7-labelled active β1 integrins colocalized with endogenous Rab7, especially at peripheral protrusive regions, where both endogenous Rab7 and active integrins accumulated (Fig. 6). Given the implication of ERC1 in focal adhesion disassembly (Fig. 4) and the involvement of Rab7 in active integrin internalization^15^ and focal adhesion turnover^16^, we tested if ERC1 could target Rab7-positive endosomes at focal adhesions. TIRF imaging on living cells cotransfected with mCherry-ERC1, cerulean-Zyxin, and GFP-Rab7 showed the accumulation of the 3 proteins at the periphery of the ventral plasma membrane of COS7 cells plated on fibronectin (Fig. 7a and Supplementary movie S5). Interestingly, high resolution imaging showed that the 3 proteins partially colocalized near zyxin-positive focal adhesions, each with a specific pattern of distribution. Time-lapse analysis showed that the ERC1-positive platforms around focal adhesions were relatively stable, while Rab7-positive endosomes were dynamic and mobile, and zyxin-positive adhesions included both stable focal adhesions and more dynamic nearby zyxin-positive structures that overlapped with some of the mobile Rab7-positive endosomes (Fig. 7b and Supplementary movie S6). Silencing of either ERC1 or liprin-α1 induced a clear increase in the concentration of Rab7-positive clusters at the periphery of the ventral plasma membrane, as detected by dynamic TIRF analysis of COS7 cells cotransfected with GFP-Rab7, mCherry-Zyxin and either control, ERC1, or liprin-α1 siRNA (Fig. 7c and Supplementary movie S7). Quantification normalized for the total projected cell area that is known to be reduced after ERC1 or liprin-α1 silencing (Fig. 7d), confirmed the increased accumulation of the Rab7-positive endosomes and of zyxin-positive focal adhesions at the periphery of cells silenced for either ERC1 or liprin-α1 (Fig. 7e–g and Supplementary Fig. S4). No increase of either Rab7-positive vesicles or focal adhesions was detected in the central basal area of these cells, indicating that the concentration at the cell periphery was a specific consequence of the silencing of ERC1 or liprin-α1 proteins. Overexpression of either the liprin-N or the liprin-ΔN mutant did not evidently affect the distribution of Rab7-positive vesicles at the cell periphery (Supplementary Fig. S5). Interestingly, quantitative analysis on TIRF images showed that ERC1 depletion specifically increased the concentration of Rab7 around peripheral focal adhesions, but not near central focal adhesions, suggesting a specific role of ERC1 in the trafficking of Rab7 endosomes in proximity of dynamic peripheral focal adhesions (Fig. 7h).

To test the specificity of the localization of Rab7 at focal adhesions we have compared the distribution of Rab6 in cells transfected with a plasmid coding for GFP-Rab6. Rab6 regulates the transport and targeting of exocytic carrier vesicles, and in HeLa cells ERC1 localizes to patches that function as sites for preferential targeting of the Rab6-positive vesicles^17^. In COS7 cells the distribution of GFP-Rab6 was clearly different from that of GFP-Rab7, with evident concentration of GFP-Rab6 in the perinuclear area (Supplementary Fig. S6). In contrast to GFP-Rab7, no significant effects were observed on the distribution of GFP-Rab6 in cells depleted of ERC1 (Fig. 7i,j), indicating that ERC1 specifically affected the peripheral accumulation of Rab7. Moreover, neither Rab4, Rab5, nor Rab11 GTPases involved in endocytic traffic from/to the plasma membrane^18^,^19^ showed any specific enrichment around active β1 integrins at protrusions (data not shown). Based on these findings we hypothesize that ERC1 is required for the Rab7-dependent internalization of active integrins to promote the disassembly of focal adhesions.

## Discussion

We have addressed the role of the liprin-α1 and ERC1 in the regulation of cell edge dynamics by using both protein silencing and deletion mutants affecting liprin-α1 homo-dimerization and the recruitment of ERC1 at the cell edge. We have prepared deletion constructs to identify the amino-terminal region of liprin-α1 that is required for the formation of liprin-α1 homo-dimers. The fragment including the first 344 amino acid residues of liprin-α1 is sufficient and necessary for binding to other liprin-α1 molecules (Fig. 1).

The requirement of the identified amino-terminal fragment of liprin-α1 to mediate the homotypic interaction with other liprin-α1 molecules suggests that liprin-N may act as a dominant negative form that binds endogenous liprin-α1 and interferes with the formation of normal homodimers of endogenous liprin-α1, thus preventing the formation of the endogenous functional complexes required for cell motility. This hypothesis was supported by the observation that the expression of the liprin-N fragment inhibited tumor cell motility and invasion, as previously shown after knocking down either liprin-α1 or one of its functional partners ERC1 and LL5^10^. Neither frequency nor persistence of the lamellipodia were affected in cells expressing the liprin-N mutant. Therefore the inhibition of cell migration after liprin-N expression is due to a reduced protrusive capacity that is independent from the ability of the cells to form lamellipodia *per se*. Notably, expression of the liprin-N mutant induced an elongated cell shape characterized by narrower protrusive areas, which differ from the larger lamellipodia observed in cells overexpressing full length liprin-α1, and could account for the impaired migratory behaviour of the liprin-N-expressing cells.

A defect in protrusive activity may originate from a defect in the turnover of focal adhesions necessary for migration on the extracellular matrix. We have recently proposed that liprin-α1 and ERC1 contribute with other scaffold proteins to the formation of PMAPs placed at specific sites of the plasma membrane^11^, where they regulate a number of diverse dynamic processes such as synaptic transmission, extracellular matrix remodelling, and cell migration^6^,^20^,^21^. Liprin-α1 and ERC1 belong to dynamic and polarized PMAPs that form near focal adhesions at the front of different types of migrating cells^9^,^10^,^22^. These proteins localize also at the cortex of non-motile HeLa cells where they form platforms to anchor microtubules^8^,^23^. Recent experimental evidence supports a role of PMAPs in the dynamics of focal adhesions. Silencing of either liprin-α1 or the ERC1-binding partner LL5β by specific siRNAs induces larger focal adhesions^10^,^22^. Moreover silencing of liprin-α1 increases the lifespan of focal adhesions, while overexpression of either liprin-α1 or ERC1 decreases their lifespan^10^,^24^. Silencing of either one of two other components of PMAPs, the ERC1–interacting protein LL5β and the LL5β-associated microtubule-interacting protein CLASP, also increases the size and lifetime of focal adhesions, and decreases their disassembly rate^22^. Here, we have established a role of endogenous ERC1 in supporting the turnover of focal adhesions by specifically promoting the disassembly of adhesions at the periphery of migrating cells. The data show that either ERC1 silencing or the expression of the liprin-N fragment, which may interfere with the formation of the endogenous liprin-α1 homo-dimers and the localization of endogenous functional complexes, have negative effects on the dynamics of focal adhesions, resulting in longer-lived, more static adhesions. A dominant negative effect of liprin-N is supported by the mis-localization and diffusion away from the cell edge of either overexpressed or endogenous ERC1 observed in cells expressing liprin-N.

Membrane traffic influences focal adhesion dynamics by internalizing and recycling integrin receptors. Both inactive (low affinity for extracellular ligands) and active integrin receptors (high affinity for ligands) can be found on the cell surface, and depletion of either liprin-α1 or ERC1 specifically inhibits the internalization of active β1 integrins^10^. Interestingly, confocal and TIRF analysis have shown here a differential colocalization of distinct subsets of active integrin β1 clusters either with ERC1 or with classical cytoplasmic focal adhesion components (Fig. 5). The alternative colocalization suggests the hypothesis that ERC1 may lead to focal adhesion disassembly by replacing cytoplasmic focal adhesion components to favour the internalization of the active receptors, thus leading to the disassembly of part of the adhesive structure.

Analysis of the involvement of distinct populations of endosomes in integrin traffic has shown that Rab7-positive endosomes are specifically involved in the internalization of active β1 integrins^15^. Interestingly, we observed a specific concentration of Rab7-positive endosomes near focal adhesions at the periphery of spread COS7 cells and at the leading edge of MDA-231 cells migrating on fibronectin. It was previously reported that active β1 integrins are specifically internalized in Rab7-positive endosomes^15^. In this study was shown that active β1 integrins are rapidly internalized, while inactive β1 endocytosis is neutralized by rapid recycling to the plasma membrane, resulting in inactive β1 integrin being largely at the cell surface, while active β1 is mainly intracellular. Here we found that endogenous Rab7 was concentrated and often colocalized together with internalized active β1 integrins at the front of MDA-231 cells, indicating intense polarized internalization of active β1 integrins in Rab7-positive endosomes during migration. Given the specific effects of liprin-α1 and ERC1 in focal adhesion disassembly, and the requirement of these proteins for efficient active β1 integrin internalization, it may be hypothesized that the liprin-α1/ERC1 functional complexes regulate the disassembly by favouring the internalization of active β1 integrin into Rab7-positive endosomes. In this direction, depletion of either endogenous ERC1 or liprin-α1 caused an increase of the concentration of Rab7-positive endosomes at the cell periphery. Based on these findings we propose a model, whereby the inhibition of the disassembly of focal adhesions induced by silencing either ERC1 or liprin-α1 protein may reflect a defect of active β1 internalization in Rab7-positive endosomes.

**Figure 8:**
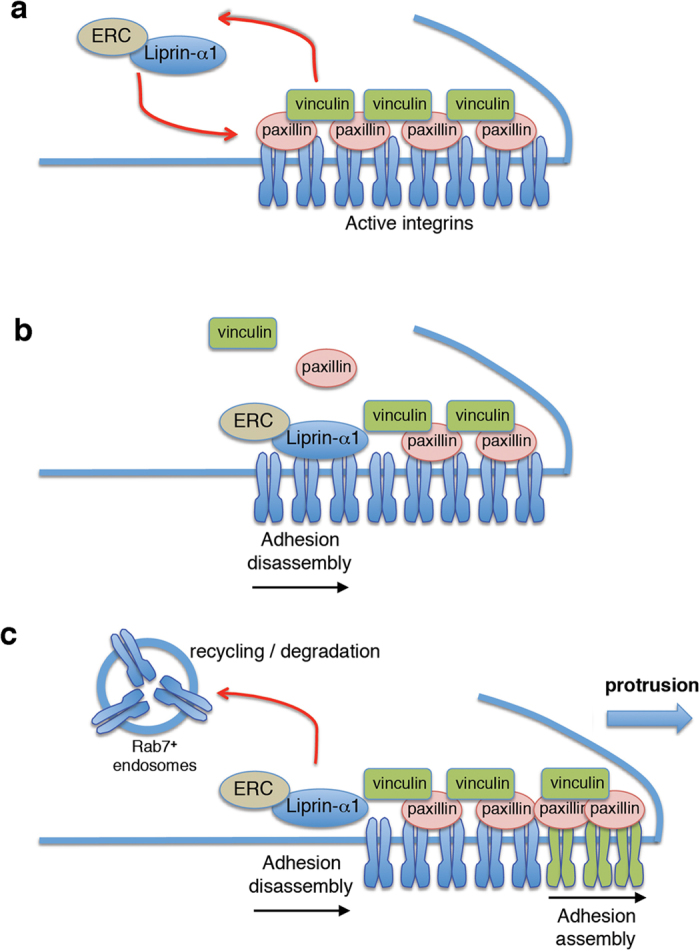
Model for the role of the liprin-α1 and ERC1 on focal adhesion turnover. During migration, liprin-α1 and ERC1 are recruited together at focal adhesions near the cell edge (**a**) displacing its cytoplasmic components (**b**), to facilitate the internalization of active integrins into Rab7-positive endosomes and promote focal adhesion tunover and protrusion (**c**). A defect in the recruitment of liprin/ERC functional complexes prevents efficient focal adhesion disassembly inhibiting protrusion.

We would like to propose a model supported by the inhibition of focal adhesions disassembly by ERC1 depletion and by the negative effects of the liprin-N and liprin-ΔN homodimerization mutants on adhesions dynamics. According to this model (Fig. 8), the specific recruitment of liprin-α1/ERC1 at the cell edge regulates the disassembly of focal adhesions and the internalization of active β1 integrins into a Rab7–positive compartment. The lack of proper localization of liprin-α1–ERC1 at the cell edge would prevent the disassembly of peripheral focal adhesions, negatively affecting their turnover and causing an increase in their size. This would eventually cause a defect in migration.

In summary, we have shown that the functional interaction of liprin-α1 and ERC1 is important to regulate the dynamics at the edge of migrating cells, by regulating the disassembly of focal adhesion to allow protrusion at the cell front. The data support a model in which liprin-α1 and ERC1 belong to PMAPs assembling dynamically around focal adhesions, to promote adhesion disassembly by sequestering active integrin β1 receptors to be internalized. Interfering with the formation of proper liprin-α1 homo-complexes and with the localization of ERC1 may represent efficient ways to interfere with cell migration, and with invasive tumor cell motility in particular. Liprin-α1 belongs to the liprin family of scaffold proteins, and is highly expressed in human breast and other tumors^2^,^25^. The family includes liprin-α and -β proteins that form homo- and hetero-oligomers, and may interact with a number of other protein partners^26^. Therefore liprins may contribute to the formation of large assemblies linking together structural and signaling proteins^4^,^5^. In neurons ERC and liprin-α cooperate in the assembly of presynaptic scaffolds needed for efficient synaptic vesicle release^27^,^28^. We propose that in non-neuronal cells the functional interaction of liprin-α1 with ERC1 is important for the assembly of platforms involved in the turnover of focal adhesions at the front of migrating cells.

## Methods

### Plasmids, siRNAs and transfection

Plasmids for full length human FLAG-Liprin-α1, GFP-Liprin-α1, FLAG-Liprin-1-670. and FLAG-Liprin-333-1009 were as described^29^,^30^. Plasmids for the Liprin-α1 mutants were described in the Supplementary Method section. Plasmids for mCherry-Zyxin and Cerulean-Zyxin were from dr. Victor Small, mCherry-ERC1 was as described^10^, and GFP-Rab6 and GFP-Rab7 plasmids were from dr. Marino Zerial.

The siRNA for human liprin-α1 and for Luciferase were as published^2^,^24^. Three siRNAs were used for human ERC1a, ERC1-1, ERC1-2^10^ and ERC1-3 (target sequence: 5′-CTGAAGGAAGTATTAAGAGAA-3′, from Qiagen). All siRNAs (50 nM) were transfected using Lipofectamine 2000 (Thermo Scientific) in serum-free medium. Cells were incubated in growth medium for 1 or 2 days after transfection before biochemical or functional assays. All siRNAs efficiently down-regulated the endogenous proteins.

### Cell lines and antibodies

COS7 and HeLa cells were cultured in DMEM with 10% fetal clone III (Hyclone), or 10% fetal bovine serum (Euroclone), respectively. MDA-231 cells were growth in DMEM/F12 1:1 with 10% fetal bovine serum.

The antibodies used in this study include: anti-liprin-α1 polyclonal antibody (pAb)^24^; anti-liprin-α1 pAb (Proteintech) and mAb (SantaCruz); anti-ERC1a mAb (ELKS-30) and Rab7 pAb (Abcam); mAb 9EG7 specific for active β1 integrins and anti-paxillin (clone 349; BD Biosciences); mAbs anti-tubulin-α (Clone DM1A) and anti-FLAG (M2), and pAbs anti-FLAG and anti-vinculin (Sigma); anti-GFP rabbit pAb (Thermo Scientific) and chicken pAb (Abcam). Alexa-Fluor–conjugated secondary antibodies (Thermo Scientific).

### Generation and characterization of stable cell lines

The human breast cancer MDA-231 cell line used in this study has been authenticated by DNA profiling by the use of a service provided by Identicell (MOMA, Aarhus, Denmark). Plasmids for the tagged liprin-α1 mutants GFP-Liprin-N and GFP-Liprin-ΔN were transfected into MDA-MB-231 cells with Lipofectamine-2000 (Life Technologies). Cell lines obtained by limiting dilution in medium with 1 mg/ml G418 (Merck Millipore) were tested for the expression of the liprin mutants by immunofluorescence and immunoblotting, and for viability by the MTT assay. For the MTT assay, 2000 cells/well were seeded in 96 well plates and viability was measured every 24 h. Statistical analysis was performed by one-way ANOVA and Dunnett’s Multiple Comparison Test. Selected clones were then used for functional analysis as described in the Results.

### Functional assays and cell shape analysis

For random migration, MDA-231 cells were plated, transfected and acquired as previously described^2^. The analysis of the frequency and persistence of lamellipodia was performed on frames from time-lapses for random migration assays according to a published protocol^10^. For morphological analysis, projected cell area, circularity, and aspect ratio were measured in transfected migrating MDA-231 cells using the ImageJ software.

The circularity was calculated as: 4π x projected cell area/cell perimeter^2^ with 1.0 corresponding to a perfect circle, and lower values predicting an elongated shape. The aspect ratio was calculated as the ratio: major cell axis/minor cell axis.

For Matrigel (BD Transduction) invasion assays, stable cell lines expressing either GFP, GFP-tagged wild type liprin-α1, or liprin-α1 mutants were used, as described^10^.

### Immunoprecipitation

Immunoprecipitation from cell lysates was performed as described^24^. Briefly, antibody pre-adsorbed to 25 μl of protein A Sepharose beads (Amersham Biosciences) was added to lysates (200–300 μg protein/immunoprecipitation), and incubated for 3 h at 4 °C with rotation. Immunoprecipitates were washed in lysis buffer with inhibitors for proteases (0.3 mM PMSF and Complete, from Roche) and phosphatases (10 mM NaF, 1 mM NaV), and separated by SDS-PAGE for immunoblotting.

### Immunofluorescence and image analysis

MDA-231 cells plated at low density on fibronectin-coated coverslips were used for immunofluorescence after 1 day, as described^29^. Briefly, cells were fixed for 10 min with 3% paraformaldehyde at room temperature, permeabilized with 0.1% saponin in PBS, incubated 2 h with primary Abs, washed, incubated with secondary Abs, and kept in PBS for acquisitions by TIRF, or mounted with ProLong Gold antifade mounting solution (Thermo Scientific) for confocal microscopy. For the analysis of the differential colocalization of active β1 integrins with either ERC1 or vinculin, images were captured using a TIRF (H2 Nikon STORM-TIRF Eclipse TI) microscope with a 1.49 NA/100X oil objective (Nikon) equipped with a back-illuminated CCD camera (Andor iXon3 DU-897). Quantification was made on areas defined by a distance of 0.8 μm all around each vinculin-positive focal adhesion. For the localization of Rab7-positive vesicles at/near zyxin-positive focal adhesions, images were captured using a TIRF (3D TIRF-GSD) microscope with a 63x/1.47 NA oil immersion objective (Zeiss) at 90 nm of penetration. Images adjusted for brightness and contrast were converted to binary images after background subtraction. Resulting binary masks of 5 × 4 μm areas around vinculin-positive focal adhesions in each channel were processed using ‘Image Calculator’ of ImageJ. Integrated density values from the resulting images were measured, averaged and plotted into the graphs.

For the analysis of Rab-positive vesicles, COS7 cells were plated at low density (40.000 cells/well) on 2.5 μg/ml fibronectin-coated 24 mm diameter coverslips, and cotransfected with siRNAs, mCherry-Zyxin, and either GFP-Rab6 or GFP-Rab7. Two days after transfection cells were fixed and acquired using a TIRF microscope (3D TIRF-GSD, Leica) with a 63x/1.47 NA oil immersion objective (Zeiss) at 90 nm of penetration. Identical exposure and laser power were used for all samples. For the analysis of Rab-positive vesicles, quantification of the area occupied by Rab staining was performed with ImageJ on thresholded images. The same threshold was used for all conditions within each experiment. The resulting masks for each channel were used to measure the area occupied by vesicles and by focal adhesions (identified by zyxin, vinculin or paxillin) in the total and in the central area of the cell, defined as the portion of the projected cell area described by a closed line equidistant 10 μm from the cell edge. The area of cell periphery occupied by the Rab signal or by focal adhesions was evaluated as the difference between the signal in the total cell area, and the signal in the central area of the cell. Each value was normalized to the cell area. For quantification, 3–4 independent experiments were used.

### Live imaging and focal adhesion turnover

For live cell imaging, COS7 cells transfected with GFP-Rab7 were recorded starting 48 h after seeding at low density. During the acquisition with an inverted TIRF (3D TIRF-GSD, Leica) microscope, cells were incubated at 37 °C and 5% CO_2_ in a controlled stage incubator (Oko-Lab), and kept in phenol red-free DMEM medium (Thermo Scientific) with 10% FBS. Movies were acquired with an APO 1.47 NA/63X oil objective (Zeiss) and an evanescent field depth of ~90 nm.

Live imaging of MDA-231 cells was performed on an inverted laser scanning confocal microscope (TCS SP8 SMD-FLIM, Leica). Cells plated at low density on 35 mm diameter fibronectin-coated (2.5 μg/ml) glass bottom dishes (MatTek) were cotransfected either with siRNAs and mCherry-Zyxin, or with GFP-Liprin-α1 mutants and mCherry-ERC1, and recorded 24–48 h after transfection. Live imaging was performed on an inverted laser scanning confocal microscope (TCS SP8 SMD-FLIM, Leica). During the acquisition cells were maintained in phenol red-free DMEM medium (Thermo Scientific) with 10 % FBS, at 37 °C and 5% CO_2_ using a controlled stage (Oko-Lab). A white light laser lane in combination with HyBrid detectors were used to maximise the sensitivity of the photon detection and to minimize phototoxicity. Images were acquired with a 63x/1.4 NA oil immersion lens (Zeiss) for 1 h at 1 frame/min. Same exposure times and laser power were used for all the samples. Quantitative analysis of the density of focal adhesions was performed with ImageJ, on thresholded images of mCherry-Zyxin. The same exposure times, laser power and threshold were used for all conditions within each experiment. Quantification was performed on the first image of the time series. Data were pooled from 2–3 independent experiments.

Focal adhesion turnover was estimated by a modification of a published protocol^31^,^32^, on kymographs created by tracing a line of 10 μm along the axis of growth of the adhesions. The assembly rate was the rate of extension of the distal adhesion tip towards the cell edge, and was calculated as the ratio between the extension in pixel (ΔD) and the time of extension (ΔT) (Supplementary Fig. S7). The disassembly rate was the rate of retraction of the proximal adhesion tip, and was calculated as the ratio between the retraction in pixel (ΔD) and the time of retraction (ΔT). The halt time was the period a focal adhesion was stable, without any assembly or disassembly event. The lifespan was the length of time a focal adhesion was visible during the time of acquisition (1 h).

### Internalization of active β1 integrins

MDA-231 cells were cultured for 24 h on fibronectin-coated (2.5 μg/ml) 24-mm coverslips, then incubated for 1–2 h at 37 °C in growth medium with 1.25 μg/ml 9EG7 mAb. After fixation and permeabilization with 0.1% saponin, cells were immunostained with Alexa-Fluor-conjugated secondary antibodies. Images were acquired with an UltraViewer spinning disk confocal microscope.

### Statistical analysis

Significant differences were evaluated by one-way ANOVA and Dunnett’s multiple comparison test, or Student t-test (* or °*p* < 0.05; ** or °°*p* < 0.005; *** or °°°*p* < 0.0005).

## Additional Information

**How to cite this article**: Astro, V. *et al*. Liprin-α1 and ERC1 control cell edge dynamics by promoting focal adhesion turnover. *Sci. Rep.* **6**, 33653; doi: 10.1038/srep33653 (2016).

## Supplementary Material

Supplementary Information

Supplementary Movie 1

Supplementary Movie 2

Supplementary Movie 3

Supplementary Movie 4

Supplementary Movie 5

Supplementary Movie 6

Supplementary Movie 7
