# Supplementary material for: Liprin-α1 and ERC1 control cell edge dynamics by promoting focal adhesion turnover
Source: Sci Rep. 2016 Sep 23;6:33653. doi: 10.1038/srep33653 (PMC5034239; doi:10.1038/srep33653)
Supplement: Supplementary Information [file srep33653-s1.pdf]

SUPPLEMENTARY INFORMATION

i. Supplementary Methods

ii. Supplementary Figure 1-8

iii. Supplementary Movie 1-7

Liprin-α1 and ERC1 control cell edge dynamics by promoting focal adhesion turnover

Veronica Astro[1], Diletta Tonoli[1], Sara Chiaretti[1], Sabrina Badanai[1], Kristyna Sala[1], Marino Zerial[2], Ivan de Curtis[1*]

[1]Cell Adhesion Unit, Division of Neuroscience, IRCSS San Raffaele Scientific Institute and San Raffaele University, via Olgettina 58, 20132 Milano, Italy.

[2]Max-Planck-Institute of Molecular Cell Biology and Genetics, Pfotenhauerstr. 108, 01307 Dresden, Germany.

*Corresponding author: Ivan de Curtis, Division of Neuroscience, San Raffaele Scientific Institute and San Raffaele University, Via Olgettina 58, 20132 Milano, Italy. Email: decurtis.ivan@hsr.it

Supplementary Methods

The FLAG-Liprin-N (residues 1-344) was obtained by digestion of pFLAG-Liprin-1-670 plasmid with Hind III, and subcloning into pFLAG-CMV2 (Kodak) and pGFP-N1 vectors (Clontech laboratories, MountainView, CA). FLAG-Liprin-1-517 was obtained by PCR on the FLAG-Liprin-1-670 with primers 5'-GGAATTCCATGAT GTGCGAGGTGATGC-3' and 5'-CGCGGATCCTTATAGTTCAGCCCTCAGTGCT-3', and subcloning after digestion with EcoR I and BamH I into pFLAG-CMV2. FLAG-Liprin-ΔN (lacking residues 1-334) was obtained by PCR from FLAG-Liprin-α1 with primers 5'-GGAATTCCATGGAAGCCACATCTGTGCATG-3' and 5'-GGAATTCTTAGCAGGAGT

AAGTCCTGA-3'. The PCR product digested with EcoRI was subcloned into pFLAG-CMV2 and pGFP-C1 vectors. FLAG-Liprin-ΔN1 (Δ1-140) was obtained by PCR on FLAG-Liprin-α1 with primers 5'-GGAATTCCATGACCGTGGTGAAGAGAC-3' and 5'-GGAATTCTTAGCAGGAGTAAGTCCTGA-3'. FLAG-Liprin-ΔN2 (Δ1-215) was obtained by PCR on FLAG-Liprin-α1 with primers 5'-GGAATTCCATGCTA ACAGATGGAGTGCTGG-3' and 5'-GGAATTCTTAGCAGGAG TAAGTCCTGA-3'. The PCR products digested with EcoR I were subcloned into pFLAG-CMV2 vector.

Supplementary Figures

Supplementary Figure S1. Effects of liprin-ΔN on random migration. MDA-231 cells transfected with the indicated constructs and siRNAs were quantified for the speed of migration (n=5 experiments).

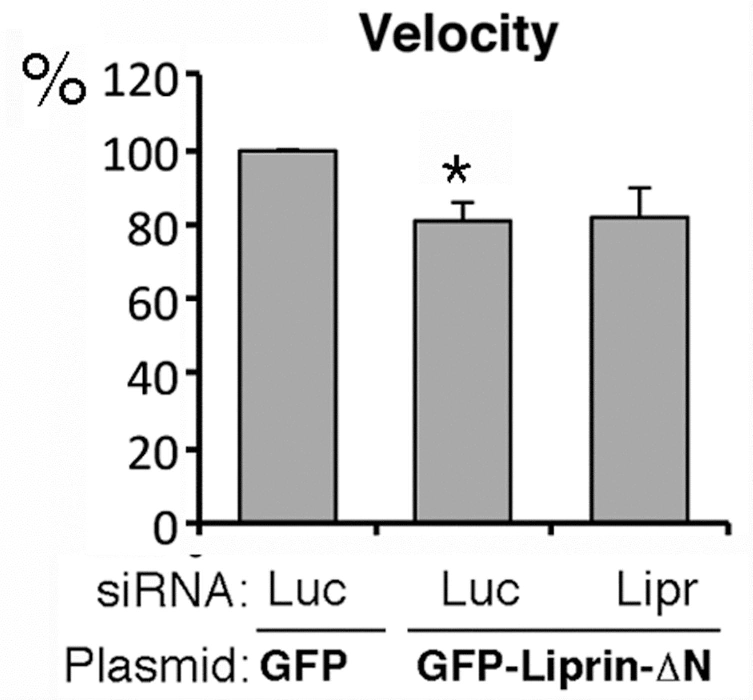

Supplementary Figure S2. Downregulation of endogenous liprin-α1 in cells cotransfected with siRNA and liprin-ΔN. MDA-231 cells cotransfected with siRNA for liprin-α1 and the indicated GFP plasmids were lysed; 30 μg of protein lysate was loaded on each lane for immunoblotting with anti-liprin-α1 antibodies. The lower filter was incubated with anti-tubulin antibody.

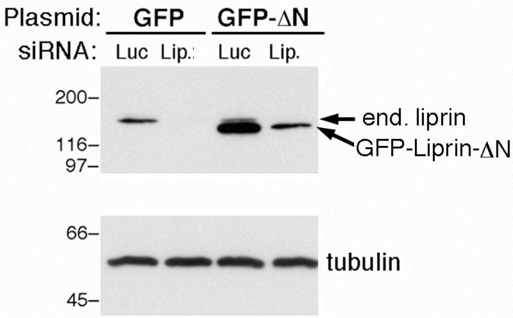

Supplementary Figure S3. Cell proliferation is not affected in stable cell lines expressing liprin-α1 mutants. Cell viability was measured by the MTT assay as described in the Methods. No significant differences were detected among stable clones expressing either GFP, GFP-N, or GFP-ΔN, and wild type MDA-231 cells.

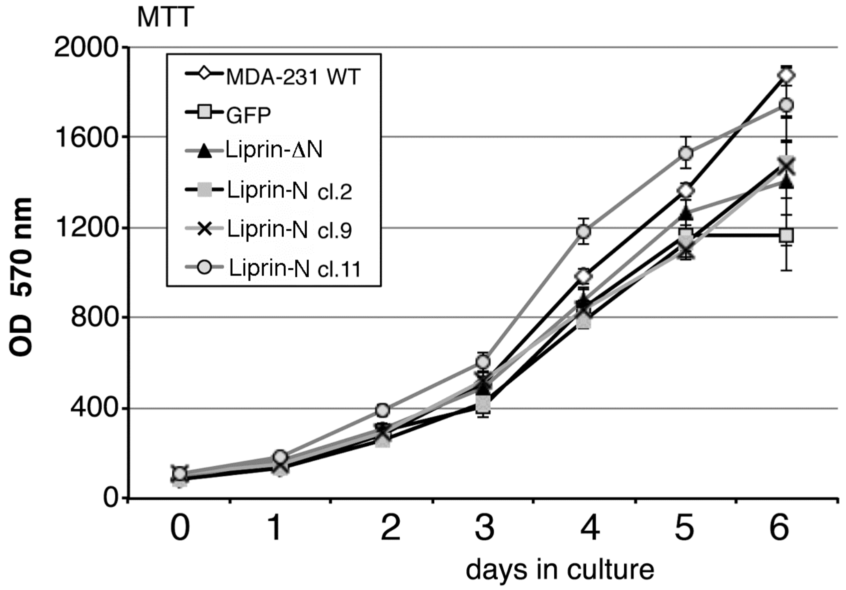

Supplementary Figure S4. Silencing of endogenous liprin-α1 and ERC1 by specific siRNAs. Immunoblotting on lysates of MDA-231 cells transfected with the indicated siRNAs, and blotted with either anti-liprin-α1 (left) or anti-ERC1 (right) antibodies. Tubulin was used as a control for loading.

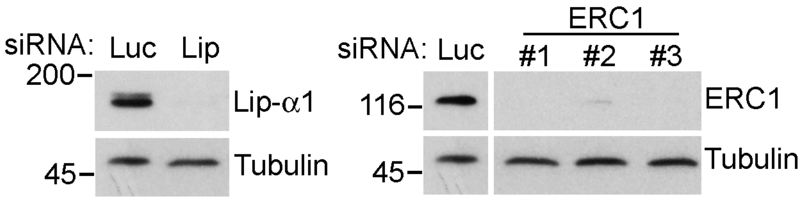

Supplementary Figure S5. Expression of either liprin-N or liprin-ΔN does not affect the distribution of Rab7. Quantification of the distribution of Rab7 in COS7 cells cotransfected with GFP-Rab7, Cerulean-Zyxin, and either FLAG-βGalactosidase, FLAG-Liprin-N, FLAG-Liprin-ΔN, or FLAG-Liprin-α1. Triple-transfected cells were analyzed by TIRF microscopy to quantify the distribution of Rab7, as described in **Fig. 7e**. Values were normalized to control βGalactosidase-transfected cells. No significant differences between cells transfected with either liprin construct and βGalactosidase were detected.

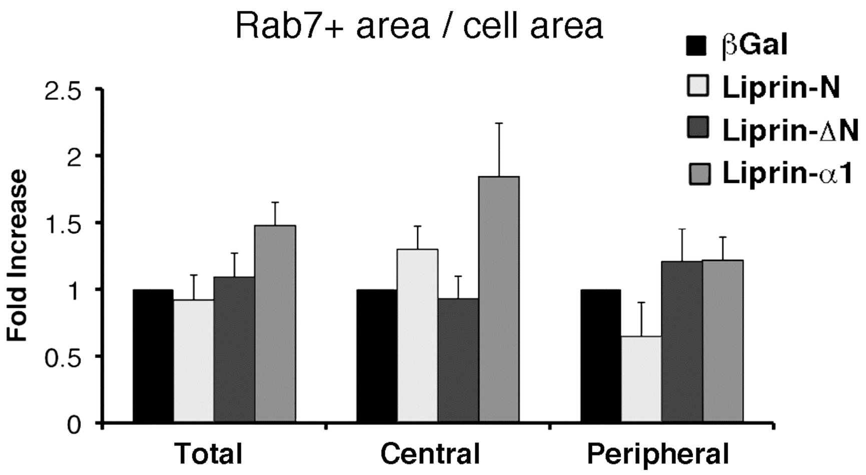

Supplementary Figure S6. Distribution of Rab7 and Rab6. Confocal microscopy of COS7 cells transfected with either GFP-Rab7 or GFP-Rab6, and immunostained for GFP, endogenous ERC1, and paxillin. Scale bar, 20 μm. Enlargement (scale bar, 6 μm) of the boxed areas in the upper panel.

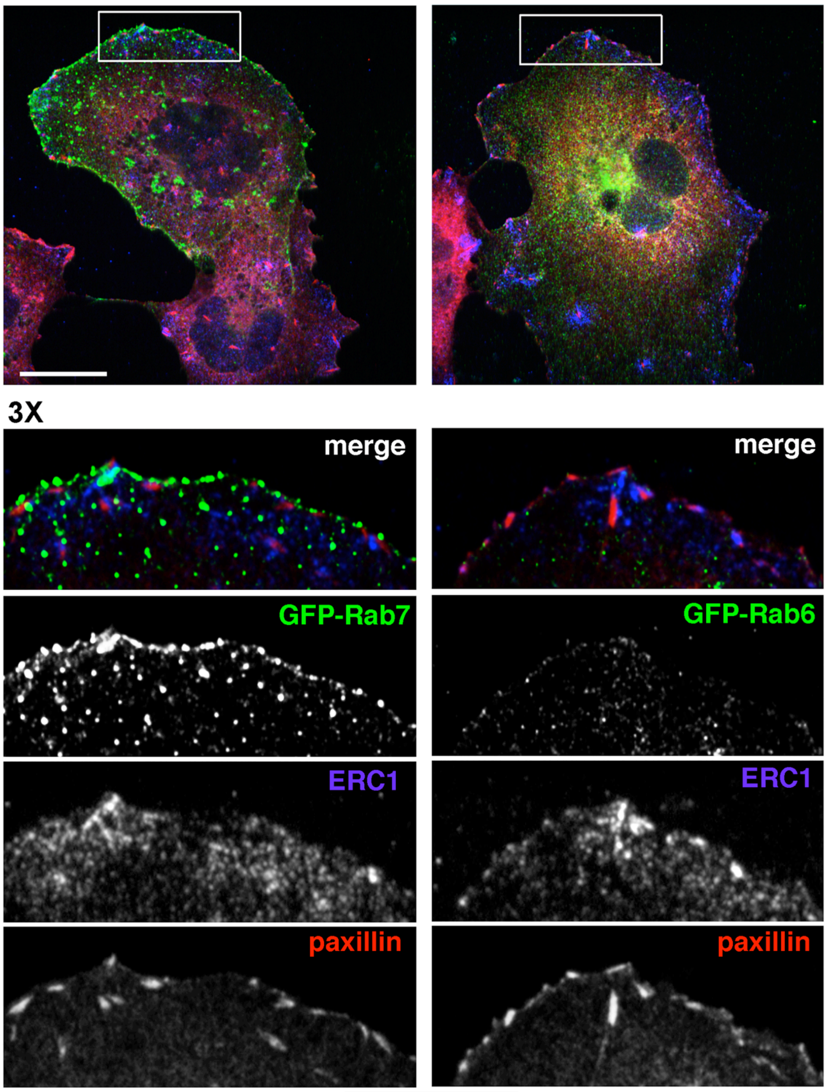

Supplementary Figure S7. Focal Adhesion Analysis on Kymographs. (**a**) Time-lapse showing mCherry-Zyxin positive focal adhesion assembly (upper panel), disassembly (central panel), and halt (lower panel) during 15 min acquisition. Scale bar, 2 μm. (**b**) Kymographs of the focal adhesions shown in (**a**) was created by plotting the time (15 min) along the x axis and distance along the y axis. The slopes of the white dotted lines in the upper and central panels indicate the rates [= distance (ΔD) x time^{-1} (ΔT)] of focal adhesion assembly and disassembly, respectively. See Methods for details.

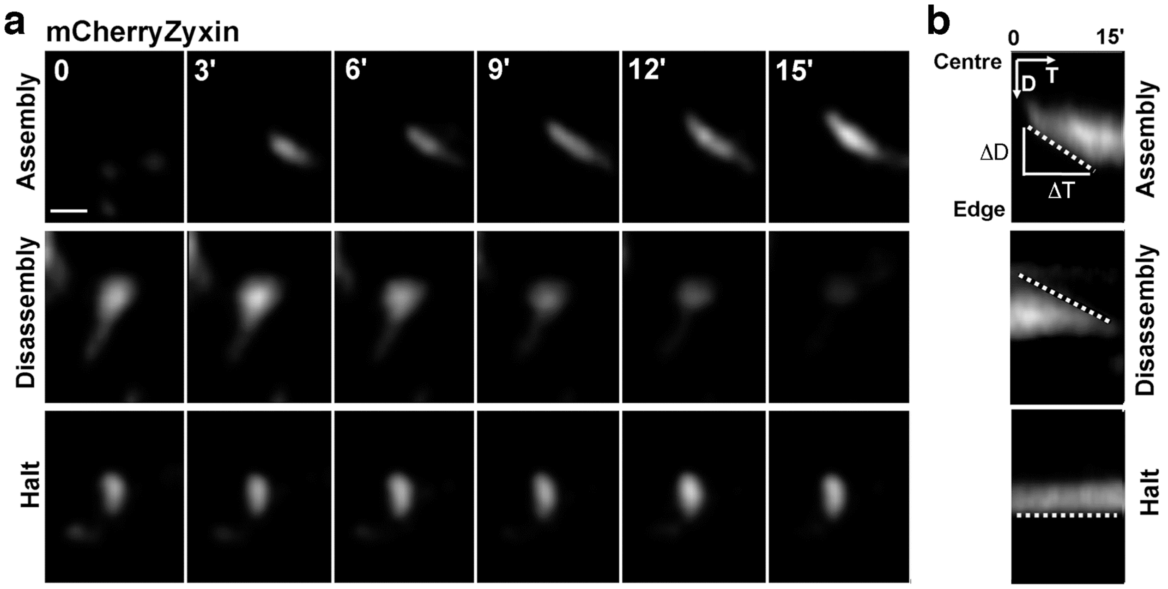

Supplementary Figure S8. Full blots. (**a**) Full blots of cropped blots shown in **Fig. 1c,d**. (**b**) Full blots of cropped blots shown in **Fig. 2a,d**. (**c**) Full blots of cropped blots shown in **Fig. 4a**.

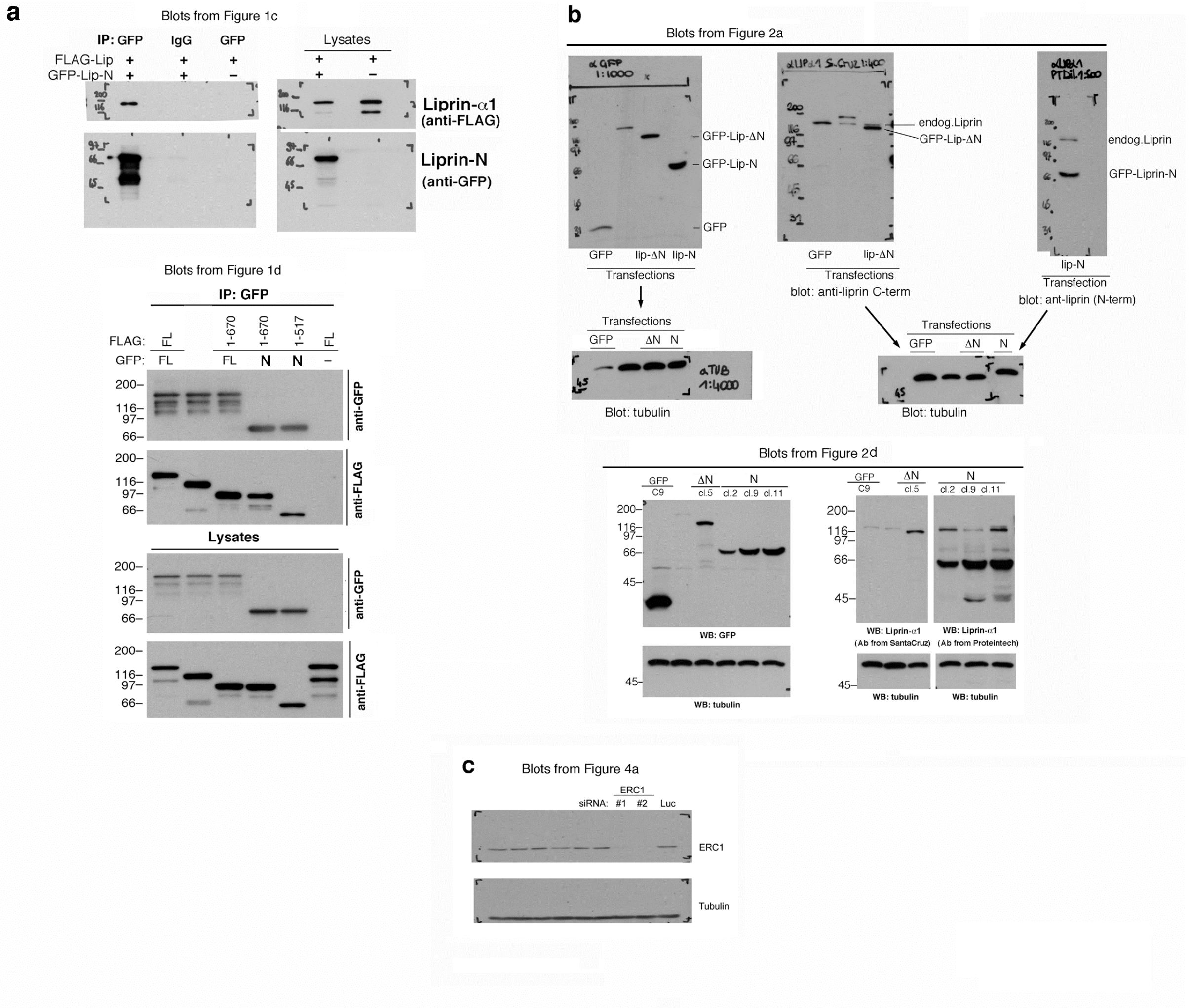

Supplementary Movies

Supplementary Movies S1-S4. Effects of the expression of different liprin-α1 constructs on the dynamics of focal adhesions. MDA-231 cells were cotransfected with mCherry-Zyxin together with GFP (**Movie S1**), GFP-Liprin-α1 (**Movie S2**), GFP-Liprin-N (**Movie S3**), or GFP-Liprin-ΔN (**Movie S4**). Time-lapses were acquired 48 h after transfection for 60 minutes at one frame every min, using a Leica TCS SP8 Scanning Confocal microscope equipped with a HC PL APO CS2 1.40 NA/ 63x oil objective. Scale bar, 10 μm.

Supplementary Movie S5. Localization of Rab7 and ERC1 at focal adhesions. Time-lapse of COS7 cell on fibronectin-coated coverslips, coexpressing GFP-Rab7, mCherry-ERC, and Cerulean-Zyxin. TIRF images were taken every 15 seconds, using a SR GSD 3D TIRF Leica microscope with a 1.47 NA/ 63x oil objective, and an evanescent field depth of 90 nm. Scale bar, 20 μm.

Supplementary Movie S6. Localization of Rab7 and ERC1 at focal adhesions. This movie is an enlargement of a the time-lapse shown in **Movie S2**. Scale bar, 5 μm.

Supplementary Movie S7. Localization of Rab7 in cells silenced for either ERC1 or liprin-α1. Live TIRF imaging (evanescent field depth of 90 nm) on COS7 cells plated on fibronectin-coated coverslips and cotransfected with GFP-Rab7, mCherry-Zyxin and siRNAs for either control (left), ERC1 (middle), or liprin-α1 protein (right). The time-lapses were performed using a GSD TIRF Leica microscope with a 1.47 NA/ 63x oil objective, equipped with a Andor XUltra camera. Cells were recorded for 3 min, with one frame taken every 2 sec. The lower panels are the inverted lut of the green channel (GFP-Rab7), and show the

mislocalization and the defective dynamics of the Rab7-positive vesicles in ERC1 (middle) and liprin-α1 (right) depleted cells. Scale bar, 20 μm.
